# Towards a Neurocognitive Profile of Post-Acute COVID-19: A Longitudinal Study Integrating Subjective Complaints and Computerized Cognitive Testing

**DOI:** 10.3390/brainsci16090964

**Published:** 2026-09-12

**Authors:** Miroslava Hristova, Radka Massaldjieva, Penka Atanassova, Daniela Kantareva

**Affiliations:** 1 Department of Neurology, Faculty of Medicine, Medical University of Plovdiv, 4002 Plovdiv, Bulgaria; penka.atanasova@mu-plovdiv.bg; 2 Clinic of Neurology, University Hospital for Active Treatment “Sv. Georgi” (UMBAL “Sv. Georgi”), 4000 Plovdiv, Bulgaria; 3 Department of Healthcare Management, Faculty of Public Health, Medical University of Plovdiv, 4002 Plovdiv, Bulgaria; radka.massaldjieva@mu-plovdiv.bg; 4 Department of Nursing Care, Faculty of Public Health, Medical University of Plovdiv, 4002 Plovdiv, Bulgaria; daniela.kantareva@mu-plovdiv.bg

**Keywords:** long COVID, post-COVID condition, cognitive impairment, CogState, attention, neuropsychological assessment, cognitive reserve

## Abstract

**Highlights:**

**What are the main findings?**

- Attention/psychomotor impairment was the most persistent objective cognitive deficit, affecting approximately two-thirds of participants at both assessments.
- Longitudinal change appeared domain-specific: selected learning/working-memory measures improved nominally, although these changes did not survive correction for multiple comparisons, and baseline subjective complaints did not predict cognitive performance at follow-up.

**What are the implications of the main findings?**

- Subjective symptom screening alone may not adequately identify persistent objective cognitive impairment in post-COVID patients.
- Objective, repeatable computerized cognitive assessment may improve the longitudinal evaluation of post-COVID cognitive dysfunction.

**Abstract:**

**Background/Objectives:** The relationship between subjective cognitive complaints and objective performance in post-acute COVID syndrome (PACS), and the persistence of objective impairment over extended follow-up, remain unclear. This prospective longitudinal study characterized the neurocognitive profile of PACS by integrating computerized cognitive testing with structured evaluation of subjective complaints across an extended post-infection interval. **Methods:** A total of 102 adults with a history of COVID-19 infection (3–35 months post-infection; mean age 40.2 ± 10.95 years; 70.6% female) underwent baseline (V1) CogState Battery testing (seven subtests) and structured assessment of persistent post-COVID complaints; 67 participants (65.7%) constituted the matched longitudinal sample and completed the follow-up (V2) after a mean inter-visit interval of 6.69 ± 1.00 months. Domain-specific composites (Attention/Psychomotor function, Learning/Working memory, and two Global indices) were derived from age-adjusted z-scores, with impairment defined as z ≤ −1.5. **Results:** Persistent complaints were reported by 63.7% of participants at baseline. In the matched longitudinal sample (*n* = 67) Attention/Psychomotor impairment was the most prevalent and stable finding in the paired sample (65.7% at V1 and 67.2% at V2; McNemar *p* = 1.000), whereas ONB-defined working-memory impairment declined nominally in the paired sample (39.4% to 24.2%; *p* = 0.006), although this change did not remain significant after correction for multiple comparisons (BH-FDR q = 0.063). In the baseline sample persistent complaints were associated with poorer objective performance at V1 across all composite indices with small-to-moderate effect sizes (all BH-FDR-adjusted q = 0.042–0.047). These associations were not observed in the follow-up sample at V2. Age was the most robust demographic correlate of impairment, particularly for visuospatial associative memory (CPAL; β = −0.349, *p* < 0.001); clinical and demographic predictors explained 9.4–13.9% of the variance (R^2^ = 0.094–0.139) in significant models at Visit 1. **Conclusions:** Attention/psychomotor dysfunction constituted a persistent objective deficit after COVID-19. Subjective complaints at baseline were associated with objective impairments but did not predict cognitive performance at follow-up. These findings support the use of objective computerized cognitive testing alongside symptom screening in the clinical assessment of post-COVID patients. Interpretation is limited by the absence of pre-COVID cognitive data and of a demographically matched healthy, uninfected control group.

## 1. Introduction

Post-acute COVID syndrome (PACS), also termed Long COVID or post-COVID condition (PCC), constitutes a novel clinical entity that has emerged within the context of the COVID-19 pandemic. Characterized as a chronic and markedly heterogeneous condition, PCC is estimated to affect approximately 41.79% of individuals following COVID-19 infection [1], although its true prevalence likely remains uncertain because of heterogeneous definitions, recognition and reporting.

The World Health Organization (WHO) defines PCC as new or residual symptoms manifesting within three months of acute infection, persisting for at least two months, unexplained by an alternative diagnosis, and subject to relapse or fluctuation over time, with a demonstrable negative impact on daily and occupational functioning [2,3]. Although definitions vary marginally across international bodies, all converge on the non-specific and heterogeneous nature of PCC symptomatology, its multisystemic involvement, and the absence of a validated diagnostic test [4]. The condition may follow both laboratory-confirmed and clinically probable COVID-19 infection and may develop irrespective of the severity of the acute illness—a feature that substantially complicates efforts to delineate a consistent clinical and prognostic profile [2,4,5].

Neurological and neuropsychiatric manifestations constitute a substantial component of the PCC clinical spectrum [6,7]. Headache, sleep disturbance, myalgia, hypo-/anosmia, fatigue, mood alteration, and cognitive difficulties are among the most frequently reported subjective complaints. Cognitive symptoms, frequently designated in the early pandemic literature under the umbrella term “brain fog,” typically manifest as forgetfulness, slowed ideation, impaired concentration and planning, cognitive fatigue, and a diminished capacity to manage cognitively demanding tasks. Large patient-reported cohorts have demonstrated that such subjective cognitive complaints may persist long after acute infection and substantially interfere with occupational, academic, and daily functioning [8,9].

Objective neuropsychological investigations likewise attest to a heterogeneous post-COVID cognitive phenotype, with attention, processing speed, executive function, and memory emerging as the domains most consistently implicated [10,11,12,13]. Deficits in attention and processing speed have been corroborated using both conventional paper-and-pencil measures and computerized batteries incorporating reaction-time paradigms of high measurement precision [13,14,15]. Memory findings exhibit greater variability, contingent upon the specific construct under assessment—working, associative, visual, or episodic memory. This variability bears clinical significance, since widely employed global screening instruments (e.g., the Montreal Cognitive Assessment [MoCA]) may have limited sensitivity for subtle domain-specific deficits, particularly among highly educated, young-to-middle-aged patients with otherwise preserved global cognitive functioning [16,17,18,19].

Emerging biomarker and neuroimaging findings suggest that post-COVID cognitive dysfunction may involve a combination of neurovascular, inflammatory, and functional-network abnormalities [20,21,22,23,24,25]. Blood–brain barrier disruption has been discussed and demonstrated in individuals with long COVID-associated cognitive impairment [20,21], while other studies have identified persistent inflammatory changes [20,22]. Multimodal MRI investigations have further reported metabolic abnormalities and altered white-matter integrity, supporting the hypothesis of possible persistent neurovascular and microstructural involvement [21]. Functional-connectivity studies additionally indicated disruption of cognitive networks, including the dorsal-attention, default-mode, salience-related, subcortical, and cerebellar systems, with some connectivity abnormalities correlating with executive and memory performance [23,24]. Recent metabolic-connectivity data also suggests a state of chronic hypoconnectivity of attention and executive networks in PCC, providing a potential network-level substrate for persistent attentional and executive dysfunction [25].

A central and as yet unresolved question concerns the correspondence between subjective cognitive complaints and objective cognitive performance. Several investigations have reported substantial discordance between the two [26,27,28,29,30]. In a 2022 longitudinal study, 61% of participants reported subjective cognitive complaints three months after acute COVID-19, whereas objective impairment on the MoCA was documented in only 23% [26]. Conversely, Kirchberger et al. (2023) identified objective impairment on at least one test in 55.6% of participants, while self-reported concentration and memory complaints were considerably less prevalent (24.9% and 21.9%, respectively) [31]. Similarly, Muschel et al. (2024) reported that only 49% of participants with objectively confirmed cognitive impairment concurrently endorsed subjective cognitive complaints [13]. Taken together, these findings suggest that subjective complaints and measured cognitive impairment may diverge in either direction.

Accordingly, no clear consensus has emerged regarding either the strength or the direction of this association. Some investigations report no discernible relationship between subjective and objective measures [27,28]; others describe a weak [29] or even robust association [30], while still others demonstrate a discrepancy in which subjective complaints are more prevalent than objectively measurable impairment [26]. These discrepancies most likely reflect differences in study design, demographic composition, neuropsychiatric symptom burden, and—critically—the type and sensitivity of the neuropsychological instruments employed [17,27,32].

Longitudinal evidence concerning the trajectory of post-COVID cognitive performance remains limited and inconsistent. Some cohorts exhibit partial recovery of global cognition, processing speed, memory, or executive function over time. In contrast, others display comparatively stable attention and reaction-time deficits beyond one year post-infection [14,15,33,34]. Ferrucci et al. (2022) observed improvement in selected domains between baseline and a 12-month follow-up, although residual impairment remained common [33]. Schild et al. (2024) likewise reported improvement across memory/learning, executive function, attention, and language at 12-month follow-up [27]. By contrast, Martin et al. (2024) and Hennemann et al. (2024) described relatively stable attention-related deficits over time [15,34]. More recent longitudinal data suggest that cognitive recovery may be most pronounced early in the follow-up interval, whereas longer-term trajectories appear heterogeneous, with subsequent improvement, relative stability, or persistent impairment varying across individuals and cognitive domains [35,36,37,38,39]. In a 2025 study with a follow-up period extending to 36–42 months, Becker et al. reported pronounced improvement across some cognitive domains (e.g., memory, language, verbal fluency) and delayed or modest improvement in others, such as processing speed and executive functions, both of which remained ≥ 1 SD below the expected normative mean at 42 months. In unadjusted analyses, attention and working memory deficits showed no significant longitudinal change and remained relatively stable across the follow-up period [35]. In 2026, Maziero et al. identified four distinct 36-month trajectories for post-COVID cognitive performance: persistent unimpairment (48%), persistent impairment (32%), recovery from impairment (14%) and delayed development of impairment (7%) [36]. In the persistently impaired group, deficits were observed across all assessed domains—attention, memory, processing speed and cognitive flexibility [36]. Similarly, Björkdahl and Larsson found that cognitive deficits, particularly in attention, working memory and processing speed, remained evident at 36 months, whereas Taquet et al. demonstrated persistent and emerging cognitive symptoms 2–3 years after hospitalization [37,38].

These findings nonetheless warrant cautious interpretation, as individual pre-COVID cognitive baselines were generally unavailable. Thus, an apparent “plateau” could denote either a return to premorbid cognitive functioning or the ceiling of achievable recovery within the PCC context. Cross-study comparison is further complicated by differences in acute illness severity, timing of initial assessment, follow-up duration, choice of test battery, cohort demographics, and the thresholds applied to define impairment [15,27,33,34,35].

Computerized cognitive assessment offers a means of addressing several of these methodological limitations. Standardized administration established normative datasets, precise measurement of reaction time, accuracy, and error rate, and automated scoring collectively reduced examiner-related variability and enable more objective comparison across repeated assessments and cohorts [40,41,42]. The CogState battery is a validated computerized neuropsychological instrument assessing multiple cognitive domains, combining reaction-time and accuracy paradigms with more complex measures of associative learning and executive function. It has demonstrated favorable test–retest reliability and limited susceptibility to practice effects, rendering it particularly well suited to longitudinal designs [43,44]. Although individual CogState subtests employ distinct raw outcome metrics, the battery yields normatively standardized, transformed scores that situate results from different subtests on a common scale and permit the derivation of domain-specific composites that summarize related cognitive functions more robustly than any single measure [41].

Based on this methodological background, and despite the expanding literature data on post-COVID cognitive impairments, several salient questions remain unresolved:

1. What domain-specific cognitive deficits show the greatest persistence in the later post-COVID period?
2. What demographic and clinical factors are associated with cognitive performance across the post-COVID period?
3. To what extent do subjective cognitive complaints correspond to measured cognitive performance, and does this correspondence change over time?

Notably, comparatively few investigations have combined monitoring of cognitive functioning with computer-based testing and retesting—incorporating both continuous standardized outcomes and categorical impairment analysis—within a single cohort, and fewer still have employed the CogState battery as the primary neuropsychological instrument or included computerized cognitive assessment extending beyond twelve months after the acute infection.

The present prospective longitudinal study therefore sought to address these questions by integrating computerized cognitive assessment and reassessment using the CogState battery with structured evaluation of subjective cognitive complaints. The longitudinal comparison of cognitive performance between V1 and V2 constituted the primary objective; the analyses of demographic and clinical correlates and of the correspondence between subjective complaints and measured performance were secondary. Accordingly, we hypothesized that objective cognitive impairments would persist over the follow-up period, displaying a domain-specific pattern, and that baseline persistent subjective complaints would be associated with poorer objective cognitive performance.

## 2. Materials and Methods

### 2.1. Study Design

Our investigation was designed as an observational, prospective, longitudinal cohort study of neurocognitive complaints and objective cognitive performance in adults, assessed at least three months after laboratory-confirmed or clinically probable COVID-19 infection. Participants underwent a structured clinical interview, combined with comprehensive clinical and neurological evaluation and computerized cognitive testing at baseline (Visit 1, V1), followed by a retest assessment in a subset of participants who agreed to participate approximately six months later (Visit 2, V2). Our sample included individuals with or without persistent post-COVID complaints, enabling comparison of objective cognitive performance according to baseline complaint status. The aim was to evaluate the evolution and longitudinal changes in cognitive performance.

### 2.2. Participants and Recruitment

The study was conducted at the Neurology Clinic of UMHAT “St. George”, Plovdiv, Bulgaria, and an affiliated outpatient neurology practice (Medical Center “St. St. Kozma i Damyan”). Baseline recruitment and cognitive assessment were conducted between December 2022 and December 2023. During this period participants were recruited from three sources: (i) hospital-based sources at UMHAT “St. George”; (ii) outpatient neurological consultations; and (iii) an oral information campaign conducted among students at the Medical College, the Faculty of Medicine at Medical University of Plovdiv, as well as the wider community of Plovdiv region. All potential participants were screened for the ≥3-month post-infection eligibility criterion. Thirty-six individuals identified through Emergency department records were contacted by telephone, informed about the study aims and procedures, and invited to participate. Of these, only two agreed to participate and were enrolled. Three additional eligible Neurology clinic inpatients were identified through the hospital information system and were enrolled in the study, resulting in five participants recruited through hospital-based sources at V1.

**Figure 1:**
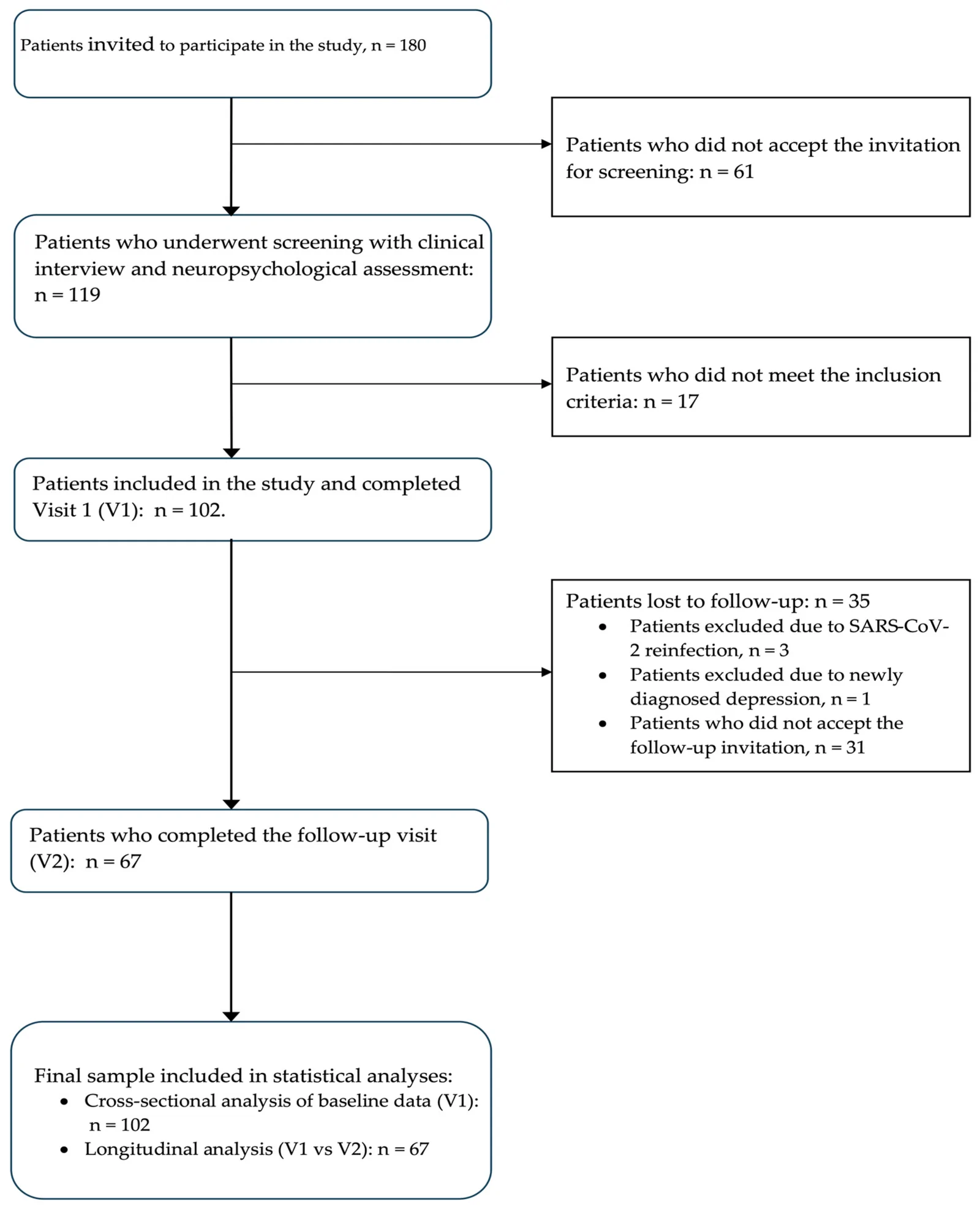
Sample flow diagram illustrating study attrition and data availability across two visits. Note: Cross-sectional analysis included all participants assessed at Visit 1 (n = 102), whereas longitudinal analysis included those participants with data from both visits (n = 67). Participants with SARS-CoV-2 reinfection or newly diagnosed depression before V2 were excluded from the longitudinal analysis.

A total of 180 individuals were invited to participate in the study, 61 of whom declined the invitation and 119 attended a screening visit. Of those screened, 17 failed to satisfy the eligibility criteria. The final sample at V1 comprised 102 participants, aged 21–60 years (Figure 1).

All visits were conducted at the two clinical sites described above. Each visit consisted of individual sessions of 60–90 min’ duration, held in a quiet setting at each participant’s convenience.

### 2.3. Eligibility Criteria

The inclusion criteria consisted of: (1) age 20–60 years; (2) history of laboratory-confirmed or clinically probable COVID-19 infection, the latter defined as a physician-recorded diagnosis based on epidemiological contact and compatible symptoms in the absence of available laboratory documentation (3) ability to provide written informed consent; (4) an interval of more than three months between acute infection and study enrolment; and (5) both sexes were eligible.

The exclusion criteria comprised: (1) history of major neurological disorders, such as acute cerebrovascular or cardiovascular events, traumatic brain injury with loss of consciousness exceeding one hour, epilepsy, malignancy, psychiatric illness, or demyelinating disease; (2) neurodegenerative disease, including but not limited to Parkinson’s disease, Alzheimer’s disease, or vascular dementia; (3) known pre-existing cognitive impairment; (4) decompensated chronic disease (cardiovascular, endocrine, renal, hepatic, pulmonary, or other); (5) history of alcohol or substance abuse; and (6) inability to complete the study.

### 2.4. Participant Characteristics and Grouping

Our sample comprised 102 Caucasian young-to-middle-aged adults of the same ethnic background, with predominantly high educational levels.

For subgroup analyses, participants were categorized into two predefined age groups (20–49 years and 50–60 years), based on the evidence that cognitive decline in otherwise healthy individuals is not typically detectable before the age of 60 [45]. Based on the presence of persistent neurocognitive complaints at baseline, participants were further classified as individuals with or without subjective post-COVID complaints. According to the interval between acute infection and baseline assessment, participants were additionally divided into three temporal categories: 3–6 months (n = 12), 7–12 months (n = 21), and >12 months (n = 69). However, these temporal strata were used for exploratory subgroup analyses and given the unequal subgroups (particularly the small 3–6-month subgroup), analyses across these categories were interpreted carefully.

### 2.5. Clinical and Demographic Assessment

At the baseline visit, all 102 participants underwent a structured clinical assessment, performed by a neurologist trained in neuropsychology. The evaluation included a predetermined questionnaire to collect demographic characteristics (sex, age), medical history, epidemiological data (time since acute COVID-19, type of test employed to confirm SARS-CoV-2 infection, need for hospitalization, vaccination status), presence of persistent post-COVID complaints, and current comorbidities. Acute COVID-19 infection was categorized according to treatment setting and oxygen requirement as outpatient management without oxygen supplementation and hospitalization requiring oxygen therapy. Comorbidity status was categorized as no known pre-existing comorbidity versus at least one pre-existing medical condition, based on available medical documentation and participants’ medical history. Persistent complaints were assessed at baseline (Visit 1) using a study-specific, clinician-administered questionnaire. Complaint status was defined as the presence of at least one reported complaint versus none. In contrast, complaint burden was represented by the total number of complaints per participant (divided categorically into four groups: 0, 1, 2 and ≥3). Emphasis was on subjective neurocognitive complaints, such as impaired memory (forgetfulness) and impaired concentration, neuropsychiatric complaints (mood changes), and functional consequences, represented by impaired occupational or everyday functioning, while insomnia, fatigue, and hyposmia were categorized as non-cognitive complaints.

All demographic and clinical findings were documented using case-report forms specifically developed for our study.

### 2.6. Procedure

#### 2.6.1. Clinical Evaluation

All participants underwent a complete somatic and detailed neurological examination. Psychiatric status was evaluated during a targeted component of the structured clinical interview, focusing on any past or present psychiatric diagnosis or current symptoms of depression, anxiety, emotional lability, or other clinically relevant psychiatric manifestations (panic attacks, psychotic or manic episodes, etc.). No standardized psychiatric questionnaires were used. The primary purpose of this interview was clinical screening to identify psychiatric factors or conditions that might influence cognitive performance and the interpretation of neuropsychological test results, rather than quantitative assessment of the severity of depressive or anxiety symptoms.

#### 2.6.2. Computerized Neuropsychological Assessment

Cognitive function was assessed using the CogState Battery (CogState Ltd., Melbourne, Australia), a computerized neuropsychological instrument developed for standardized cognitive evaluation and various combinations and subtest options for domain-specific cognitive assessment. The CogState Battery has demonstrated sound construct and criterion validity against conventional neuropsychological instruments, acceptable-to-strong test–retest reliability, and reduced susceptibility to practice effects relative to traditional paper-and-pencil tests. These properties collectively support its suitability for longitudinal designs [40,41,43,44].

For the purposes of this study, the assessment included seven CogState subtests, administered in a fixed sequence (Table 1): Groton Maze Chase Test (GMCT), Groton Maze Learning Test (GMLT), Detection Test (DET), Identification Test (IDN), One Card Learning Test (OCL), One Back Test (ONB), and Continuous Paired Associate Learning Test (CPAL). The GMCT task was administered primarily as a visuomotor familiarization task before the GMLT. Therefore, it was not included in the standardized z-scores, categorical impairment, or composite-score analyses.

#### 2.6.3. CogState Administration, Scoring and Data Quality Assessment

The CogState battery was administered using a Lenovo IdeaPad laptop (Lenovo, Beijing, China; 15.6″ display, 1920 × 1080 resolution) and a wireless computer mouse. The testing was supervised by the same examiner at V1 and V2. Before testing, all participants received standardized verbal instructions and completed the brief practice trials, incorporated into each task, according to CogState Ltd.’s recommendations.

Raw cognitive data were automatically converted and uploaded to the CogState online platform, where they were processed and transformed using the predetermined algorithms provided by CogState Ltd. Test completion and data quality were evaluated using the predefined integrity criteria provided by CogState Ltd. including minimum valid responses and accuracy thresholds. Integrity status was determined automatically following processing by the CogState platform and was therefore not available during the assessment session. Consequently, administrations failing the integrity criterion were not repeated within the same visit, as all subtests were administered consecutively in a predetermined order in accordance with CogState Ltd.’s instructions.

A distinction was made between missing test administrations, for which no numerical result was available, and completed test administrations that provided a numerical score but did not meet the corresponding integrity criterion. Therefore, missing administrations were treated as missing data, whereas completed administrations, with available numerical scores, were retained in the primary analysis.

Across all participant-level CogState test and retest sessions (*N* = 169), the platform automatically identified administrations that did not meet the integrity criteria. Such observations were infrequent (<10% overall for each subtest), generally involved isolated subtests, and were retained in the primary analyses to avoid unnecessary loss of otherwise valid data. Where the proportion of integrity failures exceeded 10% for a particular test, an additional sensitivity analysis was performed after excluding affected administrations. The aim was to assess the robustness of the main findings. Integrity status was evaluated at the level of each individual subtest administration rather than by excluding a participant’s entire battery. The extreme CPAL observation described in Section 3.8 was excluded only after contextual review of the full session identified concurrent data-quality concerns.

### 2.7. Standardized Z-Scores and Composite Cognitive Measures

The CogState transformed outcome scores from each subtest were then converted to age-adjusted standardized z-scores using test-specific, age-group-specific normative means (M) and standard deviations (SD) provided directly by CogState Ltd. in a separate normative document [46]. This source was supplemented with peer-reviewed publications describing the CogState normative framework and its application in international populations [47,48]. Bulgarian population-specific normative data were not available. Following standardization, reaction-time and error-based measures (where lower raw scores denote better performance—DET, IDN, ONB, CPAL, GMLT) were directionally reversed so that higher z-scores consistently showed better cognitive performance, and lower z-scores indicated poorer performance across all cognitive measures. Directional reversal was implemented by multiplying the corresponding standardized scores by −1; all transformations and subsequent analyses were performed in IBM SPSS Statistics version 31.0.

Age-adjusted z-scores were calculated using the following formula:

$$z=\frac{x-M}{SD}$$

where

- z = standard score (z-score).
- x = participant’s raw score on the corresponding test.
- M = mean—predetermined normative value [46].
- SD = standard deviation—predetermined normative value [46].

To provide a domain-oriented assessment of cognitive performance, we calculated composite scores using the unweighted means of the age-adjusted z-scores obtained from each CogState subtest. In accordance with established CogState methodology [41], we defined the following composites: Attention/Psychomotor function, Learning/Working memory, standard Global cognitive composite, and exploratory (extended) Global composite.

The Learning/Working memory composite was calculated as the mean of the standardized z-scores for the One Card Learning (OCL) and One Back (ONB) tests, and the Attention/Psychomotor function composite was calculated as the mean of the standardized z-scores for the Detection (DET) and Identification (IDN) tests. These two were combined in one unified measure due to their functional connection [49,50,51].

We calculated a Standard Global Cognitive composite as the mean of the age-adjusted z-scores obtained from the DET, IDN, OCL and ONB tests, providing an overall estimate of cognitive performance across the principal domain evaluated in the study. Additionally, we calculated an Extended Global Cognitive composite by averaging the standardized z-scores from the DET, IDN, OCL, ONB, CPAL and GMLT tests. The aim was to incorporate additional measures of visual associative learning and executive functions to evaluate the consistency of global cognitive findings across a broader range of cognitive domains (Table 2).

Primary cognitive impairment was defined as a composite or subtest z-score ≤ −1.5 SD, a threshold selected to balance sensitivity against specificity [52]. The applied cut-off level of ≤−1.5 SD is a commonly used criterion in neuropsychological research, especially in studies using age-adjusted z-scores.

### 2.8. Follow-Up Assessment

All 102 participants, assessed at baseline (Visit 1), were invited for a follow-up evaluation (Visit 2) six months later. The follow-up visits included consent confirmation, assessment of interval clinical events, and computerized neuropsychological assessment using the same CogState Battery sequence, testing hardware, and procedures applied at baseline. Due to participants’ personal commitments, the six-month time frame was not consistently adhered to. Therefore, the interval between visits ranged from four to nine months, with a mean duration of 6.69 ± 1.00 months (Mo = 6). However, more than half of participants (53.7%) who completed V2 were reassessed exactly six months after V1.

Of the baseline 102 participants, 67 (65.7%) completed the follow-up assessment and were included in the longitudinal analyses. The primary aim of the second evaluation was to assess changes in cognitive performance while minimizing practice effects by using a computerized neuropsychological battery specifically designed for repeated cognitive testing.

### 2.9. Statistical Analysis

Statistical analyses were performed using IBM SPSS Statistics version 31.0 (IBM Corp., Armonk, NY, USA). Statistical significance was set at α = 0.05, with results reported as *p*-values and 95% confidence intervals. Effect sizes were reported using Cohen’s d for parametric comparisons, r for Wilcoxon signed-rank analyses, and Cramer’s V for associations between categorical variables, where appropriate.

Given the low proportion of genuinely missing data (approximately 1–1.5%), no imputation was performed. Administrations with no numerical test scores were treated as missing and were handled through pairwise deletion in descriptive and univariate analyses and through listwise deletion in multivariable models. Test administrations that yielded a numerical score but failed the CogState integrity criterion were retained in the primary analysis and were not treated as missing. Sensitivity analysis was performed where integrity failures exceeded 10% of the sample. Extreme values (outliers) were not automatically excluded but were individually reviewed. One extreme V1 CPAL observation (z = −15.17) was considered unreliable because of concurrent data-quality concerns and was therefore excluded from the primary CPAL analysis. An additional sensitivity analysis retaining this observation was performed to assess the robustness of findings. Other extreme values without evidence of data-quality concerns were retained in accordance with recommendations against indiscriminate outlier removal in clinical samples [53].

Normality of continuous variables was assessed separately for each visit, using the Shapiro–Wilk and Kolmogorov–Smirnov tests, supported by inspection of histograms and Q–Q plots, where needed. Parametric tests (independent *t*-test and ANOVA) were applied to approximately normally distributed data, and non-parametric equivalents (Mann–Whitney U, Kruskal–Wallis, Wilcoxon signed-rank for paired comparisons) were used otherwise. Within-participant changes in continuous cognitive scores between V1 and V2 were assessed using paired-sample *t*-tests and Wilcoxon signed-rank tests, as appropriate. Associations between categorical variables were assessed using Pearson’s χ^2^ or Fisher’s exact test with adjusted residuals for significant associations. Relationships between continuous variables were analyzed using Pearson’s or Spearman’s correlation coefficients. Baseline complaints were analyzed both as a binary variable (absence versus presence of ≥1 complaint) and by symptom burden. Total complaint count was used in Spearman correlation analysis to assess ordinal/monotonic associations, whereas four complaint-count categories (0, 1, 2, ≥3) were used in analyses of categorical cognitive impairment. Independent associations between selected demographic and clinical factors and cognitive performance were evaluated by using multiple linear regression. Changes in the frequency of categorical cognitive impairment between V1 and V2 were assessed using McNemar’s test for participants with valid paired data for the respective measure.

Given the exploratory nature of the study, Benjamini–Hochberg false discovery rate (BH-FDR) correction was applied separately within coherent families of related analyses to account for multiple testing. Adjusted q-values were calculated for the principal longitudinal categorical and continuous comparisons, baseline complaint–composite comparisons, and the additional sensitivity analyses examining the influence of the exact inter-visit (V1–V2) interval.

### 2.10. Ethical Considerations

The study was approved by the local Research Ethics Committee of the Medical University of Plovdiv (Protocol No. 5/17.06.2022) and was conducted in accordance with the principles of the Declaration of Helsinki (2013). All participants provided written informed consent prior to enrolment. Data were used exclusively for research purposes and were accessible only to members of the research team.

## 3. Results

### 3.1. Sample Flow and Attrition Analysis

The study sample comprised 102 COVID-recovered adults at baseline. Of these, 67 (65.7%) completed the planned follow-up evaluation approximately 6.69 ± 1.00 months after V1. A total of 35 patients were lost to follow-up due to various reasons, such as newly diagnosed depression (n = 1), SARS-CoV-2 reinfection (n = 3), or personal engagements and unavailability at the predefined follow-up window (n = 31). The participants’ flow through the study is presented in Figure 1.

Participants who did not complete the follow-up assessment (n = 35) did not differ significantly from those retained (n = 67) in sex, age, time since COVID-19 infection, or the presence of baseline complaints, nor in most baseline categorical cognitive indices (all *p* > 0.05). In particular, impairment in the primary Attention/Psychomotor function composite was comparable between non-followed-up and retained participants (71.4% vs. 65.7%; *p* = 0.555).

Participants lost to follow-up did, however, exhibit a lower baseline prevalence of comorbidities (20.0% vs. 43.3% among those retained; *p* = 0.019) and a higher frequency of impaired GMLT performance (14.3% vs. 0%; Fisher’s exact *p* = 0.004) (Table 3). Given the small absolute number of participants with impaired GMLT performance, this finding should be interpreted cautiously. This pattern may indicate a degree of attrition-related selection, which should be considered when interpreting the longitudinal findings.

However, overall attrition analysis was not associated with substantial differences in most baseline characteristics, although potential selection related to comorbidities and GMLT performance cannot be excluded.

The proportion of OCL administrations that failed the integrity criterion at V2 exceeded 10% (11.9%). Therefore, we conducted a sensitivity analysis, repeating all statistical tests involving OCL and OCL-derived composites, while excluding participants who failed the integrity criterion for OCL at Visit 2. This reduced the OCL-analytical sample from 67 to 59 participants. Following their exclusion, the mean OCL z-score increased from 0.416 ± 1.217 to 0.681 ± 0.991. The findings concerning sex, persistent complaints, and OCL-derived composites remained unchanged. The age-group OCL performance also remained statistically significant. It showed a slightly larger effect size in the sensitivity analysis (*p* = 0.013, Cohen’s d = 0.741) than in the primary analysis (*p* = 0.014, Cohen’s d = 0.680). These findings support the robustness of the principal continuous-score and composite analysis. However, no participants met the categorical OCL impairment threshold after the integrity-failed administrations were excluded. Therefore, caution is warranted when interpreting the prevalence of categorical OCL impairment at Visit 2.

### 3.2. Sample Characteristics

The mean age of our sample was 40.21 years (mean = 40.21; SD = ±10.95, min–max 21–60), and women were predominant with 70.6% (n = 72). Most participants had completed more than 12 years of education (n = 99; 97.1%), with the following educational levels: Bachelor’s/Master’s in 89.2% (n = 91), 7.8% (n = 8) were finishing their degree at the time of testing, and 3 (2.9%) had a lower formal education.

The mean time between the acute COVID-19 infection and baseline assessment was 15.23 months (mean = 15.23, SD = ±7.58), with intervals varying between 3 and 35 months (Mo = 12). The preponderance of individuals (n = 94, 92.2%) had experienced mild to moderate COVID-19 infection managed in the outpatient clinic with no need for oxygen supplementation. Less than half of the participants (45.1%, n = 46) were vaccinated. Approximately one third of the sample (35.3%, n = 36) had at least one pre-existing comorbidity, with arterial hypertension being the most common (17.6%, n = 18). The remaining 64.7% had no known chronic medical conditions.

The baseline demographic and clinical characteristics of the sample are summarized in Table 4.

The demographic and clinical characteristics of the participants who completed the follow-up assessment remained comparable to the baseline sample. At V2, the mean interval between acute infection and retesting was 21 months (mean = 21; SD = ±7.2). The proportions of women, participants with high education, and individuals managed at outpatient clinics remained similar to those observed at baseline.

### 3.3. Persistent Subjective Post-COVID Complaints at Baseline

Persistent post-COVID complaints were reported by 65 of 102 participants (63.7%) at baseline. Nearly half of the sample (48.1%) reported two or more concurrent complaints, and the presence of ≥3 concurrent symptoms constituted the most frequent pattern among symptomatic participants (26.5% of the full sample). Subjective complaints were more frequent among women (68.1% vs. 53.3% among men) and within the 50–60 years age group (74.1% vs. 60% in the younger 20–49 age group). However, there was no statistically significant association between the presence of subjective post-COVID complaints and factors such as sex (*p* = 0.159), age groups (*p* = 0.192) and pre-existing comorbidities (*p* = 0.375). Complaint frequency also did not differ significantly across the three time-since-infection strata (3–6, 7–12, and >12 months).

Similarly, complaint burden was not significantly correlated with continuous cognitive scores at either visit. However, at V1, categorical impairment for Learning/Working memory (*p* < 0.001), and both Global composites (standard *p* = 0.021 and exploratory *p* = 0.040) differed significantly across complaint-count groups, with higher impairment among participants reporting two complaints. CPAL impairment showed a similar descriptive pattern, but the overall association did not reach statistical significance (*p* = 0.062). Linear-by-linear analyses did not support a monotonic increase in impairment across complaint-count categories, indicating that this association is non-linear and not strictly burden-dependent, likely reflecting a group-specific rather than dose–response pattern.

Forgetfulness and impaired concentration were the most commonly reported neuropsychiatric complaints, followed by mood changes and difficulties in occupational or everyday activities (Table 5).

### 3.4. Standardized Cognitive Performance and Categorical Impairment

Mean z-scores were negative for nearly all CogState measures at both visits (except for OCL and GMLT), indicating overall below-normative performance across the sample. The most pronounced deficits, evident both in mean z-score and in the proportion of participants meeting the impairment threshold (z ≤ −1.5), were observed for CPAL, as well as DET, IDN, and their composite, the Attention/Psychomotor function (Table 6).

At V1, Attention/Psychomotor composite showed impairment in 67.6% of participants (69/102)—the highest rate among all indices examined—compared with 9.9% for Learning/Working memory, 34.7% for the standard Global index, and 32.0% for the exploratory Global index. The mean scores for Attention/Psychomotor composites were lower, suggesting poorer cognitive performance, within the older 50–60 years age group, among women, among participants with concurrent comorbidities, and in the 3–6-month post-acute interval. The frequency of categorical impairment confirms greater prevalence of impairment in women, within the 50–60 age group and among those with pre-existing comorbidities. Attention/Psychomotor impairment was descriptively more frequent in the >12-month group than in the 3–6-month-group (72.5% vs. 58.3%). However, the impairment in the Attention/Psychomotor composite did not differ significantly by sex (*p* = 0.100), age group (*p* = 0.615), pre-existing comorbidity status (*p* = 0.361) or time since infection across the three predefined strata (3–6, 7–12, and >12 months, *p* = 0.776).

At V2, the overall pattern remained largely unchanged: Attention/Psychomotor impairment persisted as the most prevalent finding (67.2%, 45/67), whereas the two Global composites exhibited numerically lower impairment rates (27.3% and 15.2%, respectively) and CPAL impairment declined from 38.6% to 30.3%.

McNemar tests conducted on paired data (n = 65–67, depending on the measure) showed no categorical change that remained statistically significant after BH-FDR correction. The largest nominal change was observed for ONB. In the paired sample with available ONB data (n = 66), impairment decreased from 39.4% (26/66) to 24.2% (16/66) at V2 (unadjusted McNemar *p* = 0.006; BH-FDR q = 0.063). Eleven participants transitioned from impaired to unimpaired status, compared with one participant in the reverse direction. The ONB is therefore considered exploratory.

**Figure 2:**
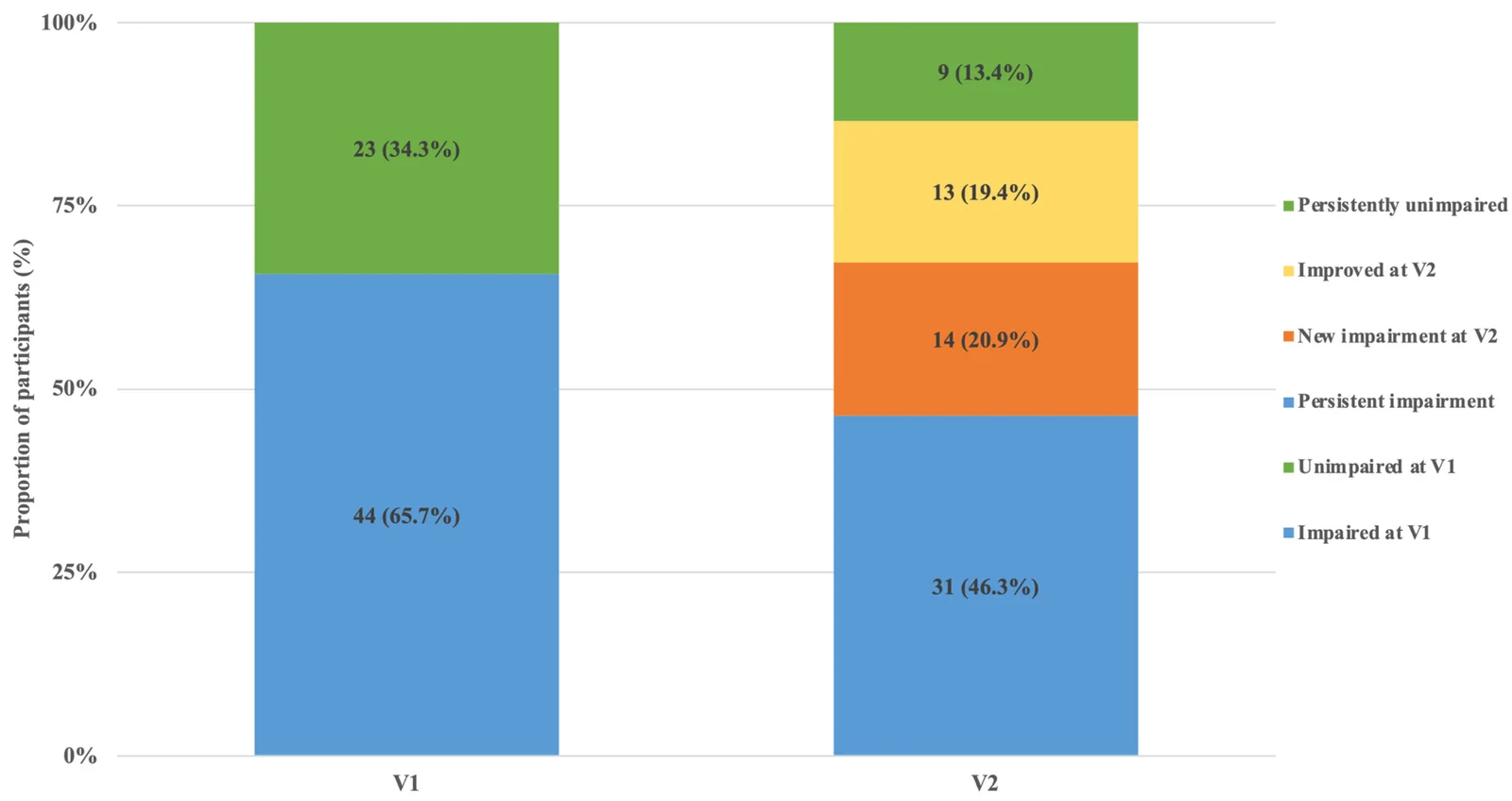
Longitudinal transitions in Attention/Psychomotor Function impairment between Visit 1 and Visit 2. Note: The stacked V1 bar shows the overall proportions of impaired and unimpaired participants at baseline, while the V2 bar shows transition in impairment status between visits. Only participants who completed both assessments are included (N = 67). Cognitive impairment was defined as an age-adjusted z-score ≤ −1.5. Persistent impairment indicates impaired performance at both visits; new impairment at V2 indicates unimpaired performance at V1 followed by impairment at V2; improvement at V2 indicates impaired performance at V1 followed by unimpaired performance at V2; and persistently unimpaired indicates unimpaired performance at both visits. At V2, the two lower segments together represent participants classified as impaired (45/67; 67.2%), whereas the two upper segments represent participants classified as unimpaired (22/67; 32.8%). McNemar’s test showed no significant change in impairment prevalence between the two visits (*p* = 1.000).

With specific regard to the Attention/Psychomotor composite, 46.3% of followed participants (31/67) exhibited impairment at both visits, 20.9% (14/67) developed new impairment by V2, 19.4% (13/67) demonstrated improvement, and only 13.4% (9/67) remained unimpaired throughout—indicating that persistent impairment in this domain characterized nearly half of the longitudinal subsample (Figure 2).

Unexpectedly, at V2 there was a slight shift in the mean z-score results, with lower scores registered among men, as well as within the younger (20–49 years) age group and participants with no pre-existing comorbidities. Similarly to V1, impairment in Attention/Psychomotor function did not differ significantly at V2 based on sex (*p* = 0.172), age group (*p* = 0.480) and pre-existing comorbidities (*p* = 0.728).

### 3.5. Within-Participant Longitudinal Changes in Cognitive Performance

Paired statistical analyses were conducted among participants with valid cognitive data in both visits (n = 65–67 depending on the subtest) to evaluate within-participant changes in continuous age-adjusted z-scores. No significant changes were observed for DET, IDN, OCL, ONB, GMLT, the Attention/Psychomotor function composite, and the Standard Global Cognitive index (Table 7). Thus, most cognitive outcomes remained statistically stable between V1 and V2.

A small nominal improvement was observed for CPAL performance (Wilcoxon signed-rank *p* = 0.031; r ≈ 0.27), with higher z-scores at V2 in 40 participants. However, this finding did not remain statistically significant after BH-FDR correction (Supplementary Table S7). The Learning/Working memory composite also showed a small nominal improvement between visits (mean difference, MD = V1 − V2 = −0.172; unadjusted *p* = 0.042; Cohen’s d = −0.257), but this observation did not survive BH-FDR. Neither of its components (ONB and OCL) showed significant continuous-score change. The Exploratory Global Cognitive index approached but did not reach statistical significance (*p* = 0.060; r ≈ 0.23).

In contrast, the Attention/Psychomotor Function composite remained essentially unchanged on continuous analysis (mean difference, MD = V1 − V2 = 0.031; *p* = 0.782; Cohen’s d = 0.034), consistent with the absence of a significant change in categorical impairment prevalence, reported in Section 3.4, thus confirming the stable pattern over time. For ONB, the continuous-score change did not reach statistical significance (*p* = 0.082). The reduction in the proportion of participants meeting the categorical impairment threshold was nominally significant (McNemar *p* = 0.006) but did not survive BH-FDR correction (q = 0.063).

A sensitivity analysis was performed, and the exact V1-V2 interval was examined in relation to longitudinal cognitive change. Longer inter-visit intervals showed nominal positive correlation with changes in DET (ρ = 0.254, *p* = 0.038), Attention/Psychomotor function (ρ = 0.256, *p* = 0.036), the Standard (ρ = 0.317, *p* = 0.010) and Extended Global composites (ρ = 0.274, *p* = 0.027). However, none of these remained statistically significant after BH-FDR correction (all q ≥ 0.095, Table S6).

Similarly, although several effects in the supplementary repeated-measures linear models reached nominal significance, none remained statistically significant after BH-FDR correction. Overall, the analyses did not provide robust evidence that variation in follow-up duration influenced the longitudinal findings.

### 3.6. Age-Related Differences in Cognitive Performance

When comparing the two pre-defined age groups (20–49 vs. 50–60 years), significant age-related differences were observed in selected cognitive measures. CPAL z-scores demonstrated significantly poorer performance in the older group at both visits (V1: *p* = 0.002; V2: *p* = 0.013; small-to-moderate effect sizes, respectively, r ≈ 0.30 and r ≈ 0.31), consistent with the higher prevalence of the CPAL categorical impairment observed descriptively in the older group at V1 (20/27, 74.1% in participants aged 50–60 years vs. 19/74, 25.7% in participants aged 20–49 years).

Performance was also significantly poorer in the older group at V2 (*p* = 0.014; d = 0.680), but not at V1 (*p* = 0.826). At V2, the OCL age-group difference remained significant even after excluding the administrations that failed the integrity criterion (*p* = 0.013; d = 0.741). The Exploratory Global index was likewise significantly poorer in the older group at V1 only (*p* = 0.011; r ≈ 0.26). No significant age-group differences were observed for the remaining cognitive measures.

Spearman correlation confirmed age as the demographic variable consistently associated with cognitive performance across both visits: age correlated negatively with CPAL z-scores at V1 (ρ = −0.273, *p* = 0.006) and V2 (ρ = −0.347, *p* = 0.004). No significant correlations emerged between cognitive performance and time since infection or the baseline burden of complaints at either visit.

### 3.7. Association Between Baseline Complaints and Cognitive Performance

At V1, participants without baseline complaints showed nominally better performance than those with complaints on IDN (unadjusted *p* = 0.040) and OCL (unadjusted *p* = 0.025). Participants without complaints also performed better on all four composite indices: Attention/Psychomotor (*p* = 0.047), Learning/Working memory (*p* = 0.041), standard Global index (*p* = 0.013), and Exploratory Global index (*p* = 0.027) (Table 8), with small-to-moderate effect sizes (d = 0.42–0.52). All four composite associations remained statistically significant after BH-FDR correction (q = 0.042–0.047; Supplementary Table S7). At V2, baseline complaint status was not significantly associated with any of the examined cognitive measures. Thus, the association between baseline symptoms and objective cognitive performance was evident at the concurrent baseline assessment but did not persist at follow-up.

No significant associations emerged between cognitive performance and the presence of comorbidities at either visit.

### 3.8. Multivariable Predictors of Cognitive Performance

Multiple linear regression models incorporating age, sex, presence of baseline complaints, and time since infection as predictors attained statistical significance at V1 for CPAL, the Attention/Psychomotor composite, and both Global indices, although explanatory value remained consistently modest (Table 9). Age emerged as the sole significant independent predictor of CPAL performance (β = −0.349, *p* < 0.001). For the standard Global index, sex (β = 0.216, *p* = 0.027) and presence of complaints (β = −0.224, *p* = 0.023) constituted independent predictors, with male sex and absence of complaints associated with superior performance. No individual predictor reached significance within the Attention/Psychomotor model despite overall model significance. In the Exploratory Global index model, sex emerged as a significant independent predictor, with male sex associated with higher cognitive scores (β = 0.219, *p* = 0.027). None of the regression models attained statistical significance at V2.

Regression diagnostics identified one potentially influential CPAL observation (an extreme outlier with z = −15.17; Cook’s distance = 0.306; studentized deleted residual = −5.168). Following review of the corresponding administration, the observation was considered unreliable because of concurrent data-quality concerns and was therefore excluded from the primary CPAL analysis. In the resulting model (N = 101), age remained the sole significant independent predictor of CPAL performance (β = −0.349, *p* < 0.001), and the overall model remained significant (R^2^ = 0.139, F (4,96) = 3.868, *p* = 0.006). A sensitivity analysis retaining the observation yielded a similar overall conclusion (β = −0.332, *p* < 0.001; R^2^ = 0.128).

## 4. Discussion

### 4.1. Principal Findings

This prospective longitudinal study aimed to characterize the neurocognitive profile of adults assessed during the post-acute and longer-term period after COVID-19 by integrating computerized cognitive assessment and re-assessment with structured evaluation of subjective neurocognitive complaints across an extended post-infection interval, with baseline assessment occurring 3–35 months post-infection and a follow-up assessment occurring at a mean of 21 months post-infection. Several findings merit particular emphasis. First, objective cognitive impairment remained detectable during long-term follow-up. Second, Attention/Psychomotor function constituted the most prevalent and persistent objective deficit, affecting approximately two-thirds of participants at both visits and remaining essentially unchanged over a mean inter-visit interval of 6.69 ± 1.00 months. Third, baseline subjective neurocognitive complaints were significantly associated with poorer objective performance at V1 across attention, visual memory, and all composite indices, yet this association was no longer discernible at V2. Fourth, objective cognitive performance remained largely stable over approximately six months, suggesting persistence rather than spontaneous recovery. Fifth, age constituted the most robust demographic correlate of cognitive performance, specifically for CPAL (visuospatial associative memory), and was consistently identified through comparative, correlational, and regression analyses. Finally, multivariable models accounted for only a modest proportion of the variance in cognitive outcomes, implicating unmeasured contributing factors.

### 4.2. Persistence of Attention/Psychomotor Impairment

The prevalence of Attention/Psychomotor impairment remained stable in the paired sample, increasing only slightly from 65.7% at V1 to 67.2% at V2 (McNemar *p* = 1.000). This finding stands in contrast to reports of partial recovery in global cognition, processing speed, or attention over similar or longer intervals [27,33], yet aligns with investigations describing comparatively stable attention-related deficits beyond one-year post-infection [15,34]. In our sample, Attention/Psychomotor impairment remained comparatively stable over follow-up. Impaired concentration that developed after the acute COVID-19 infection was reported by 43 participants (42.2% of the full cohort; 66.2% of the 65 participants reporting persistent complaints), providing subjective clinical context consistent with the attentional difficulties identified through objective testing. In contrast, Ferrucci et al. and Schild et al. reported improvement across several cognitive domains during longitudinal follow-up, whereas Martin et al. and Hennemann et al. observed persistence of attention-related dysfunction [15,27,33,34]. In the present sample, Attention/Psychomotor function followed the latter pattern, while the nominal improvement observed in selected learning and memory-related measures, which did not survive correction for multiple comparisons, is at most suggestive of a domain-specific rather than a uniform trajectory of recovery. This pattern is also consistent with more extended longitudinal observations. Becker et al. reported that attention remained relatively stable over 42 months, with no significant longitudinal change in unadjusted analyses, and only minimal change after adjustment, despite more pronounced improvement in several other cognitive domains [35]. Similarly, Maziero et al.’s four cognitive trajectories closely parallel the transition categories observed for Attention/Psychomotor function in our sample: persistently unimpaired, persistent impairment, improved, and newly impaired [36]. These findings further support the concept that the evolution of post-COVID cognitive performance is heterogeneous rather than characterized by a uniform pattern of recovery.

Because the CogState battery is designed to minimize practice effects through automated alternate item forms (Section 2.8), the absence of improvement across repeated testing is unlikely to reflect a ceiling artifact and more plausibly denotes a genuine, persistent deficit. This pattern is compatible with a model in which processing speed and attention—subserved by fronto-striatal and thalamo-cortical circuitry—are particularly vulnerable to post-COVID neuroinflammation and microvascular dysfunction. Although these mechanisms were not directly assessed in the current study, other studies have discussed the possible central role of persistent neuroinflammation and blood–brain barrier alterations in the pathogenesis of PCC [54,55]. Notably, ONB (working memory) was the only measure to show a reduction in impairment prevalence at the nominal level (39.4% to 24.2% impaired; McNemar *p* = 0.006), although this did not remain significant after Benjamini–Hochberg correction within the family of longitudinal categorical comparisons (q = 0.063; Supplementary Table S7). This observation is therefore reported as exploratory. It is nonetheless compatible with the possibility that recovery trajectories in PACS are domain-specific rather than uniform, in line with recent proposals of an early-plateau pattern with domain-dependent residual deficits [35,39]; confirmation would require adequately powered replication.

Continuous-score analyses similarly showed small nominal improvements in CPAL and the Learning/Working Memory composite, neither of which remained statistically significant after BH-FDR correction, together with a borderline unadjusted change in the Exploratory Global index. Despite the relatively long retest interval and CogState’s design reducing the likelihood of substantial practice effects, a limited contribution to the observed improvements in CPAL and Learning/Working Memory cannot be completely excluded. Thus, longitudinal data suggest partial improvement in selected learning/memory-related measures, while the Attention/Psychomotor dysfunction remained unchanged, supporting a non-uniform pattern of cognitive recovery in PCC.

### 4.3. The Relationship Between Subjective Complaints and Objective Performance

A central aim of this study was to examine the correspondence between subjective cognitive complaints and objective performance—a relationship reported inconsistently across the literature, ranging from substantial discordance [26,27,28] to weak [29] or strong association [30]. This controversy may find a deeper explanation if the divergent results on the relationship between cognitive complaints and test performance reported before the COVID-19 pandemic are also taken into account, for which influences have been described relating to the choice of methods, the cognitive domains included, the brain areas of interest reported, education and depression, among other factors [56]. Our baseline findings align more closely with the latter body of evidence: participants with persistent complaints performed significantly worse on IDN, OCL, and all four composite indices than those without complaints, with small-to-moderate effect sizes. This association between baseline complaint status and cognitive performance was not, however, observed at V2, even though objective impairment in the leading domain (Attention/Psychomotor function) remained highly prevalent. This may indicate that subjective complaints recorded at a single time point have limited predictive value for later cognitive outcomes. Importantly, the longitudinal analysis was designed mainly to assess whether the presence of subjective complaints at baseline was associated with subsequent objective cognitive performance. Given that subjective post-COVID symptoms may diminish, fluctuate or even resolve over time, baseline complaint status was considered as a potential clinical marker of subsequent cognitive recovery. Therefore, the absence of a significant association at V2 suggests that baseline complaint status did not identify participants with persistently poorer cognitive performance at follow-up.

One possibility is that early symptom status partly reflects effects of earlier acute-phase neuroinflammatory or affective processes that attenuate over time, while a subset of patients retains an objective deficit [54]. However, these mechanisms were not directly assessed in the present study. If corroborated in larger cohorts, this would suggest that reliance on subjective screening alone may increasingly under-detect objective impairment as the post-COVID course progresses, thereby reinforcing the value of periodic objective reassessment rather than symptom-triggered testing alone. The unexpected finding that categorical impairment is most pronounced among participants with two complaints may reflect a non-linear relationship between subjective symptom burden and objective cognitive performance. Participants with ≥3 complaints may represent a more heterogeneous group with broader symptom burden, including emotional, functional and non-cognitive complaints.

### 4.4. Age as a Vulnerability Factor

Age was the demographic variable consistently associated with cognitive performance, specifically with CPAL, across comparative (older group impaired in 74.1% vs. 25.7%), correlational (ρ = −0.273 at V1 and −0.347 at V2), and regression analyses (β = −0.349, *p* < 0.001 at V1). Because CPAL indexes visuospatial associative memory—a function independently sensitive to normal aging—this finding may reflect an interaction between post-COVID pathophysiology and age-related reductions in cognitive reserve [57] rather than a post-COVID-specific age effect per se. Disentangling these possibilities would require comparison against an age-matched, COVID-naïve control group and, ideally, pre-infection cognitive data, neither of which was available within the present design.

### 4.5. Limited Explanatory Value of Demographic and Clinical Predictors

Although regression models attained statistical significance at V1 for CPAL, the Attention/Psychomotor composite, and both Global indices, adjusted R^2^ values remained low (approximately 0.05–0.10), indicating that age, sex, complaint status, and time since infection jointly accounted for only a modest proportion of the variance in cognitive performance. This limited explanatory power is itself informative: it reinforces the view that post-COVID cognitive impairment is multifactorial in origin, with probable contributions from unmeasured variables such as acute illness severity, vaccination status, anxiety or affective symptomatology, and premorbid cognitive reserve. The loss of model significance at V2 may partly reflect diminished statistical power following attrition, as the V2 regression models included 66–67 participants compared with 101–102 at V1, rather than a genuine absence of association and should be interpreted alongside the attrition analysis presented in Section 3.1.

### 4.6. Strengths and Limitations

Several limitations warrant consideration when interpreting these findings. The sample was recruited through a mixed strategy, incorporating clinical and community sources. Nevertheless, the resulting sample may overrepresent participants with persistent complaints, and was predominantly female and highly educated, which may limit generalizability to the broader post-COVID population. Acute illness severity was not graded using a standardized validated scale (e.g., the WHO Clinical Progression Scale); ambulatory management does not necessarily equate to mild disease, which complicates severity-based comparisons. Individual infecting SARS-CoV-2 variants were not systematically established, and the study was not designed to assess variant-specific or vaccination-related differences in cognitive performance. No pre-COVID cognitive baseline was available, precluding determination of whether the observed deficits represent genuine post-COVID decline, incomplete return to premorbid functioning, or a ceiling of achievable recovery. Furthermore, because of the absence of a contemporaneous uninfected control group, the observed cognitive deficits cannot be attributed specifically to prior SARS-CoV-2 infection. The unequal distribution of participants across the predefined time-since-infection strata, particularly the small number of participants assessed at 3–6 months, limited the statistical power for subgroup comparisons and may have reduced the ability to detect performance differences according to time since infection. The low reaction-time-based scores and high prevalence of Attention/Psychomotor impairment should be interpreted cautiously, as they may represent genuine selective slowing, given their high sensitivity, but may also be associated with differences between the applied normative reference and the present population, or potential device-related timing effects. Subjective complaints were assessed only at baseline and not reassessed at V2; therefore, analyses of V2 cognitive outcomes evaluated the prospective association between baseline complaints and later cognitive performance, rather than concurrent correspondence between subjective and objective measures at follow-up. Consequently, changes in complaint status over time could not be evaluated. Furthermore, subjective symptoms were assessed through a structured clinical interview rather than validated symptom-specific questionnaires, and standardized measures of fatigue or post-COVID ‘brain fog’ were not administered. The study protocol combined a structured clinical interview with a full computerized cognitive battery, which already required considerable time and effort from participants; no further instruments were therefore added. This limits the quantitative characterization of subjective and affective symptom burden and their potential contribution to performance on computerized cognitive testing. Pre-assessment individual state variables, such as recent nicotine or caffeine consumption, time of day of testing, momentary fatigue, and psychotropic medication use, were not systematically recorded or quantified and may therefore have contributed to within- and between-participant variability in cognitive performance.

Participants lost to follow-up differed from those retained in baseline comorbidity burden and GMLT performance, indicating a degree of attrition-related selection that may bear upon the generalizability of the longitudinal findings. Although the inter-visit interval varied between 4 and 9 months, sensitivity analyses did not show a robust effect of follow-up duration on cognitive performance after BH-FDR correction. The higher prevalence of comorbidity among participants who returned for a follow-up assessment might partly reflect greater motivation for continued evaluation among those with a more complex medical history. However, this interpretation remains speculative. OCL integrity criteria were not satisfied in over 10% of Visit 2 administrations. Although sensitivity analysis supported the robustness of findings based on OCL score and OCL-derived composites, the prevalence of categorically impaired OCL performance should be interpreted cautiously, as no impaired cases remained after excluding the administration that failed the integrity criterion. Finally, comorbidity status, including thyroid compensatory status, relied upon available medical documentation rather than systematic laboratory verification in every case.

The study has several methodological strengths. The prospective longitudinal design allowed neuropsychological assessment and reassessment with the same computerized battery to evaluate changes in cognitive performance over a mean interval of approximately six months (6.69 ± 1.00 months). Baseline testing was conducted at a mean of 15.2 months (>1 year) after the acute COVID-19 infection, allowing the study to characterize persistent changes over time rather than early post-acute cognitive changes. Test administration was standardized using the same protocols, instructions, equipment, and investigator at both visits. The CogState battery provided precise measurement of reaction time, accuracy, and error-based outcomes across multiple cognitive domains. The concurrent assessment of subjective neurocognitive complaints and computerized objective cognitive performance provided clinical perspective, while both continuous performance measures and categorical impairment analyses allowed changes in average cognitive performance to be considered alongside changes in impairment status. Data completeness was high, attrition was formally evaluated, and OCL-related analyses were repeated after excluding the administrations that did not meet the integrity criterion, providing additional assessment of the robustness of our results.

### 4.7. Future Directions

Future investigations would benefit from larger and more demographically balanced cohorts, inclusion of a locally recruited healthy control group matched for demographic characteristics and tested under identical conditions, as well as standardized grading of acute illness severity, quantitative neuroimaging and neuroinflammatory biomarkers, and longer, more frequent follow-up extending beyond 24–36 months to more comprehensively characterize recovery trajectories. Designs capable of disentangling the evolving relationship between subjective symptom trajectories and objective cognitive trajectories—for instance, through repeated, parallel measurement of both constructs—would help clarify whether the decoupling observed herein reflects a genuine and clinically significant phenomenon.

## 5. Conclusions

This prospective longitudinal study characterized the neurocognitive profile of post-acute COVID syndrome by integrating computerized cognitive testing and retesting with structured assessment of subjective complaints across an extended post-infection interval. Attention/Psychomotor dysfunction emerged as the most prevalent and persistent objective deficit, remaining essentially unchanged over a mean follow-up of 6.69 ± 1.00 months despite a testing design intended to minimize practice effects. However, the absolute prevalence of categorical impairment should be interpreted cautiously, as classification was based on external age-adjusted CogState normative data and Bulgarian population-specific normative data are unavailable. Subjective cognitive complaints were common at baseline and were associated with concurrent objective impairment at initial assessment but did not significantly predict cognitive performance at follow-up. These findings suggest that subjective complaint assessment provides complementary clinical information but is not interchangeable with objective cognitive testing. Age constituted the most robust demographic correlate of impairment, specifically for visuospatial associative memory.

Nevertheless, age, together with other demographic and clinical predictors included in models, accounted for only a limited proportion of the variance in cognitive performance, underscoring the multifactorial nature of post-COVID cognitive impairment. Cognitive performance across multiple domains, particularly memory, may be influenced by age-related changes and decline. Therefore, the present design cannot clearly distinguish the long-term effects of COVID-19 from age-related changes, especially in the absence of pre-infection cognitive data and an age-matched control group. Taken together, these findings suggest that objective, repeatable computerized assessment may provide useful complementary information alongside symptom screening and traditional neuropsychological testing in the further evaluation of post-COVID cognitive complaints, given that reliance on self-report alone may increasingly under-detect persistent deficits as the post-COVID clinical course progresses.

## Figures and Tables

**Table 1:** CogState Battery subtests, cognitive domains, and outcome measures.

Subtest	Cognitive Domain	Outcome Measure
GMCT	Processing speed; visuomotor function	Correct moves per second while pursuing a moving target
GMLT	Executive function	Total errors across 5 trials learning a hidden 28-step path
DET	Psychomotor function	Speed (mean log10-transformed reaction time)
IDN	Attention	Speed (mean log10-transformed reaction time)
OCL	Visual memory and learning; attention (secondary)	Accuracy (arcsine square-root-transformed proportion correct)
ONB	Working memory; attention	Speed (mean log10-transformed reaction time)
CPAL	Visual learning and associative memory; spatial working memory	Total errors across 7 associative learning trials

Note: Higher OCL values indicate better performance, whereas lower DET, IDN, and ONB reaction times and fewer GMLT and CPAL errors indicate better cognitive performance.

**Table 2:** Structure of cognitive measures.

Composite Measure	Average of Following CogState Subtests	Cognitive Domains
Attention/Psychomotor Function	DET, IDN	Psychomotor speed, attention
Learning/Working Memory	OCL, ONB	Visual learning, recognition memory, working memory
Standard Global Cognitive Composite	DET, IDN, OCL, ONB	Global cognitive performance
Extended Global Cognitive Composite	DET, IDN, OCL, ONB, CPAL, GMLT	Broader global cognitive performance (exploratory)

Note: Composite scores were calculated as the mean of the age-adjusted standardized scores for the included CogState subtests. Higher composite scores correspond to better cognitive performance.

**Table 3:** Baseline characteristics of participants who completed versus did not complete the Visit 2 (V2) follow-up assessment.

Characteristic	Lost to Follow-Up (n = 35)	Completed V2 (n = 67)	*p*-Value
Age, years (mean rank)	49.10	52.75	0.554
Time since infection, months (mean rank)	56.81	48.72	0.189
Female sex, n (%)	23 (65.7%)	49 (73.1%)	0.435
≥1 comorbidity, n (%)	7 (20.0%)	29 (43.3%)	0.019
Baseline complaints, n (%)	22 (62.9%)	43 (64.2%)	0.895
Impaired GMLT, n (%)	5 (14.3%)	0 (0.0%)	0.004
Impaired CPAL, n (%)	13 (37.1%)	27 (40.3%)	0.757
Impaired Attention/Psychomotor, n (%)	25 (71.4%)	44 (65.7%)	0.555
Impaired Learning/Working memory, n (%)	4 (11.4%)	6 (9.1%)	0.708
Impaired Global index (standard), n (%)	15 (42.9%)	20 (30.3%)	0.207
Impaired Global index (exploratory), n (%)	15 (42.9%)	18 (27.3%)	0.112

Note: Age and time since infection were compared using the Mann–Whitney U test (mean ranks shown, as both variables departed from a normal distribution). Categorical variables were compared using the χ^2^ test or Fisher’s exact test, as appropriate. Cognitive impairment was defined as a subtest or composite z-score ≤ −1.5.

**Table 4:** Demographic and clinical characteristics of study participants at Visit 1 and Visit 2.

Characteristics	V1 (N = 102)	V2 (N = 67)
**Demographic characteristics**		
Age, years (mean ± SD)	40.2 ± 10.95	40.6 ± 11.64
Age, years (min–max)	21–60	21–60
**Sex, n (%)**		
Females	72 (70.6%)	49 (73.1%)
Males	30 (29.4%)	18 (26.9%)
**Education, n (%)**		
>12 years	99 (97.1%)	65 (97.0%)
≤12 years	3 (2.9%)	2 (3.0%)
**Acute COVID-19 characteristics**		
Time from acute infection to assessment, months (mean ± SD)	15.2 ± 7.58	21.0 ± 7.25
Time from acute infection to assessment, months (range)	3–35	7–35
Hospitalization requiring oxygen supplementation, n (%)	8 (7.8%)	5 (7.5%)
Outpatient management, n (%)	94 (92.2%)	62 (92.5%)
**Method of laboratory confirmation, n (%)**		
Rapid Antigen Test	62 (60.8%)	43 (64.2%)
Polymerase Chain Reaction (PCR)	35 (34.3%)	21 (31.3%)
Unknown or unavailable	5 (4.9%)	3 (4.5%)
Vaccinated, n (%)	46 (45.1%)	33 (49.3%)
**Comorbidity status, n (%)**		
At least one (≥1) pre-existing comorbid condition	36 (35.3%)	29 (43.3%)
No pre-existing comorbid conditions	66 (64.7%)	38 (56.7%)

Note: Data are presented as mean, standard deviation (SD), range (minimum—maximum), or number (n) and percentage, as appropriate. Percentages were calculated relative to the number of participants assessed at each visit (V1: N = 102; V2: N = 67) and may not total 100% due to rounding. The V2 column describes the 67 participants that completed the follow-up. Vaccination status refers to vaccination against SARS-CoV-2, performed before Visit 1.

**Table 5:** Frequency and profile of persistent post-COVID complaints at baseline (N = 102).

Complaint	n	% Within Complaints Subgroup (n = 65)
Forgetfulness/Memory impairment	45	69.2%
Impaired concentration	43	66.2%
Mood changes	35	53.8%
Difficulty coping with occupational duties	19	29.2%
Difficulty coping with daily activities	15	23.1%
Other non-cognitive complaints	5	7.7%

Note: Data are presented as number (n) and percentage calculated relative to participants reporting at least one persistent complaint (n = 65). Some participants reported more than one complaint; therefore, the percentages for individual complaints do not sum to 100%. Other non-cognitive complaints described were fatigue, hyposmia and insomnia.

**Table 6:** Mean z-scores and frequency of categorical cognitive impairment (z ≤ −1.5) by CogState measure at Visit 1 (V1) and Visit 2 (V2).

Measure	V1 Mean z-Score (SD)	V1 Impaired, n (%)	V2 Mean z-Score (SD)	V2 Impaired, n (%)	Difference V1 − V2, pp (95% CI)	McNemar *p*
DET	−1.891 (1.081)	61 (59.8%)	−1.890 (0.996)	42 (62.7%)	−4.5 (−19.9 to 11.3)	0.711
IDN	−1.977 (0.977)	68 (66.7%)	−1.858 (0.980)	43 (64.2%)	0.0 (−14.3 to 14.3)	1.000
OCL	0.248 (0.999)	5 (4.9%)	0.416 (1.217)	7 (10.6%)	−4.6 (−14.3 to 4.5)	0.453
ONB	−1.242 (0.969)	41 (40.2%)	−0.986 (0.956)	16 (24.2%)	15.2 (5.0 to 25.0)	0.006
GMLT	−0.004 (0.803)	5 (4.9%)	0.232 (0.787)	2 (3.0%)	−3.0 (−10.2 to 2.9)	0.500
CPAL	−1.758 (2.604)	39 (38.6%)	−1.170 (2.018)	20 (30.3%)	10.6 (−2.4 to 23.1)	0.167
Attention/Psychomotor function	−1.934 (0.887)	69 (67.6%)	−1.874 (0.903)	45 (67.2%)	−1.5 (−16.6 to 13.7)	1.000
Learning/Working memory	−0.503 (0.766)	10 (9.9%)	−0.285 (0.863)	6 (9.1%)	0.0 (−9.0 to 9.0)	1.000
Global index (standard)	−1.223 (0.687)	35 (34.7%)	−1.085 (0.670)	18 (27.3%)	3.1 (−9.9 to 16.0)	0.815
Global index (exploratory)	−1.093 (0.734)	32 (32.0%)	−0.878 (0.702)	10 (15.2) %	12.3 (0.9 to 23.7)	0.057

Note: Impairment was defined as a z-score ≤ −1.5 relative to age-adjusted CogState norms. At V1, percentages were based on N = 102 for GMLT, DET, IDN, ONB, and the Attention/Psychomotor composite; n = 101 for OCL and OCL-derived composites because one participant had no numerical OCL result; n = 101 for CPAL after exclusion of one unreliable V1 observation; and n = 100 for the Exploratory Global composite because it incorporated both OCL and CPAL. At V2, impairment percentages were based on N = 67 for DET, IDN, GMLT, and the Attention/Psychomotor composite and on n = 66 for OCL, ONB, CPAL, and remaining composites because of missing scores. McNemar *p*-values are based on paired data restricted to participants with valid results at both visits. The V1 ONB percentage shown in the table (41/102, 40.2%) refers to the full baseline sample, whereas the longitudinal comparison was based on paired ONB data (26/66, 39.4% at V1; 16/66, 24.2% at V2). Details on missing data and their handling are provided in Section 2.9 and Supplementary Table S1. For the paired longitudinal comparisons, the difference in impairment prevalence was calculated as V1 − V2 and expressed in percentage points (pp). Ninety-five percent confidence intervals for paired proportion differences were calculated using the Newcombe method; positive values indicate a reduction in impairment prevalence at V2, whereas negative values indicate an increase. Two-sided *p*-values were obtained using the exact binomial McNemar test.

**Table 7:** Within-participant changes in age-adjusted cognitive z-scores between Visit 1 and Visit 2.

Cognitive Measure	Paired, n	Statistical Test	Change Between V1 and V2 (95% CI)	*p*-Value	Effect Size
DET	67	Paired-samples *t*-test	MD = 0.081 (−0.198 to 0.361)	0.564	*d* = 0.071
IDN	67	Paired-samples *t*-test	MD = −0.019 (−0.253 to 0.214)	0.869	*d* = −0.020
OCL	65	Paired-samples *t*-test	MD = −0.163 (−0.464 to 0.138)	0.284	*d* = −0.134
ONB	66	Paired-samples *t*-test	MD = −0.172 (−0.367 to 0.022)	0.082	*d* = −0.218
GMLT	67	Paired-samples *t*-test	MD = −0.124 (−0.325 to 0.077)	0.224	*d* = −0.150
CPAL	66	Wilcoxon signed-rank test	HL = −0.342 (−0.678 to −0.035)	0.031	r ≈ 0.27
Attention/Psychomotor Function	67	Paired-samples *t*-test	MD = 0.031 (−0.191 to 0.253)	0.782	*d* = 0.034
Learning/Working Memory	65	Paired-samples *t*-test	MD = −0.172 (−0.338 to −0.006)	0.042	*d* = −0.257
Standard Global Cognitive Composite	65	Paired-samples *t*-test	MD = −0.060 (−0.212 to 0.092)	0.435	*d* = −0.097
Extended Global Cognitive Composite	65	Wilcoxon signed-rank test	HL = −0.117 (−0.256 to 0.005)	0.060	r ≈ 0.23

Note: MD represents the Mean paired difference calculated as V1-V2. Therefore, negative MD values indicate improvement at V2, whereas positive MD values indicate poorer performance at V2. For Wilcoxon signed-rank analyses, the direction of change is based on the distribution of positive and negative ranks. For paired-samples *t*-tests, MD is presented with the corresponding 95% confidence interval (CI). For Wilcoxon signed-rank analyses, HL represents the Hodges–Lehmann estimate of the paired difference (V1 − V2), with its corresponding 95% CI. Effect sizes are reported as Cohen’s *d* for paired samples *t*-tests and *r* for Wilcoxon signed-rank tests. Bold values indicate nominal statistical significance (*p* < 0.05); corresponding BH-FDR-adjusted q-values are reported in Supplementary Table S7.

**Table 8:** Comparison of composite cognitive z-scores between participants with and without baseline persistent complaints, at Visit 1 (V1) and Visit 2 (V2).

Composite Score	V1 Mean Difference	V1 *p*-Value	V2 Mean Difference	V2 *p*-Value
Attention/Psychomotor function	0.363	0.047	−0.089	0.703
Learning/Working memory	0.321	0.041	0.061	0.784
Global index (standard)	0.350	0.013	−0.005	0.977
Global index (exploratory)	n/a (M–W)	0.027	0.078	0.668

Note: Mean differences (no-complaints minus complaints group means) are presented for comparisons, using mostly independent sample *t*-test or Mann–Whitney U test (M–W), depending on distributional assumptions. Positive values indicate better performance among participants without complaints.

**Table 9:** Summary of multiple linear regression models for cognitive outcomes at Visit 1 (predictors: age, sex, presence of baseline complaints, time since infection).

Dependent Variable	N	R^2^	F (df)	*p*-Value	Significant Predictors
CPAL z-score	101	0.139	3.868 (4, 96)	0.006	Age: β = −0.349, *p* < 0.001
Attention/Psychomotor Function	102	0.094	2.514 (4, 97)	0.046	None individually significant
Learning/Working Memory	101	0.090	2.387 (4, 96)	0.056	None
Standard Global Cognitive Composite	101	0.136	3.771 (4, 96)	0.007	Sex: β = 0.216, *p* = 0.027; baseline complaints: β = −0.224, *p* = 0.023
Exploratory Global Cognitive Composite	100	0.124	3.350 (4, 95)	0.013	Sex: β = 0.219, *p* = 0.027

Note: All models included age, sex, presence of baseline persistent complaints, and time since acute infection as predictors. R^2^ values are unadjusted; adjusted R^2^ was lower across all models (0.05–0.10), indicating that the included clinico-demographic factors accounted for only a small proportion of the variance in cognitive performance. Complete regression models, including unstandardized coefficients (B), standard errors (SE), standardized coefficients (β), 95% confidence intervals for B, and *p*-values for all predictors at both visits are provided in Supplementary Table S5A,B.

## Data Availability

The data are not publicly available due to ethical and privacy restrictions, related to participant confidentiality and terms of informed consent.

## Supplementary Materials

The following supporting information can be downloaded at https://www.mdpi.com/article/10.3390/brainsci16090964/s1, Table S1: Completeness of CogState data at Visits 1 and 2; Table S2A: Predefined CogState completion and integrity criteria; Table S2B: Proportion of CogState administrations meeting the predefined integrity criteria at Visits 1 and 2; Table S3: Sensitivity analysis of OCL-related outcomes at Visit 2 after exclusion of administrations not meeting the integrity criterion; Table S4: Spearman correlations between cognitive performance and selected clinical and demographic variables at Visits 1 and 2; Table S5A: Multiple linear regression models for cognitive performance at Visits 1 and 2; Table S5B: Predictor coefficients from multiple linear regression models of cognitive performance at Visits 1 and 2; Table S6: Sensitivity analyses examining the influence of the exact inter-visit (V1–V2) interval on longitudinal cognitive change; Table S7: Benjamini–Hochberg false discovery rate-adjusted results for the principal multiple-comparison analysis families.

## Abbreviations

The following abbreviations are used in this manuscript:

ANOVA	Analysis of variance
BH-FDR	Benjamini–Hochberg false discovery rate
CI	Confidence interval
COVID-19	Coronavirus disease 2019
CPAL	Continuous Paired Associate Learning Test
DET	Detection Test
GMLT	Groton Maze Learning Test
GMCT	Groton Maze Chase Test
IDN	Identification Test
MD	Mean Difference
MoCA	Montreal Cognitive Assessment
NICE	National Institute for Health and Care Excellence
OCL	One Card Learning Test
ONB	One Back Test
PACS	Post-acute COVID syndrome
PCC	Post-COVID condition
PCR	Polymerase chain reaction
SARS-CoV-2	Severe acute respiratory syndrome coronavirus 2
SD	Standard deviation
SE	Standard error
V1	Visit 1
V2	Visit 2
VIF	Variance inflation factor
WHO	World Health Organization
