# Supplementary material for: Towards a Neurocognitive Profile of Post-Acute COVID-19: A Longitudinal Study Integrating Subjective Complaints and Computerized Cognitive Testing
Source: Brain Sci. 2026 Sep 12;16(9):964. doi: 10.3390/brainsci16090964 (PMC13604085; doi:10.3390/brainsci16090964)
Supplement: Supplementary file 1 [file brainsci-16-00964-s001.zip › brainsci-4524385-supplementary.pdf]

Supplementary files

Table S1. Completeness of CogState data at Visits 1 and 2

CogState measure	V1 available, n (%)	V1 missing, n (%)	V2 available, n (%)	V2 missing, n (%)
Groton Maze Learning Test (GMLT)	102 (100.0)	0 (0.0)	67 (100.0)	0 (0.0)
Detection Test (DET)	102 (100.0)	0 (0.0)	67 (100.0)	0 (0.0)
Identification Test (IDN)	102 (100.0)	0 (0.0)	67 (100.0)	0 (0.0)
One Card Learning Test (OCL)	101 (99.0)	1 (1.0)	66 (98.5)	1 (1.5)
One Back Test (ONB)	102 (100.0)	0 (0.0)	66 (98.5)	1 (1.5)
Continuous Paired Associate Learning Test (CPAL)	102 (100.0)	0 (0.0)	66 (98.5)	1 (1.5)

Note: Data are presented as number (n) and percentage (%) of all participants assessed at the corresponding visit (V1, N=102; V2, N=67). A value is classified as "missing" if no numerical test result was available for the corresponding subtest. Available-case analysis was used for descriptive and univariable analyses, whereas complete-case analysis was used for multivariable regression models. No imputation was performed. Test administrations with numerical results that did not meet the corresponding CogState integrity criteria were retained in the primary analysis and were not classified as "missing".

Table S2A. Predefined CogState completion and integrity criteria

CogState subtest	Standard completion count	Minimum completed responses or moves	Accuracy-based integrity criterion
Groton Maze Learning Test (GMLT)	140	140	Not specified
Detection Test (DET)	35	27	≥80% correct responses
Identification Test (IDN)	30	23	≥80% correct responses
One Back Test (ONB)	31	24	≥70% correct responses
One Card Learning Test (OCL)	80	60	≥50% correct responses
Continuous Paired Associate Learning Test (CPAL)	42	24	Not specified

Note: The completion criterion indicates the minimum number of responses or maze moves for each subtest. The integrity criteria represent the minimum accuracy thresholds predefined by CogState Ltd. Failure to meet the integrity criteria was treated as a quality indicator, not as an automatic reason for participant's exclusion. Numerical scores were retained in the primary analyses, and sensitivity analysis was performed when integrity failures exceeded 10% of administrations for a particular subtest.

Table S2B. Proportion of CogState administrations meeting the predefined integrity criteria at Visits 1 and 2

CogState measure	V1 criterion met, n (%)	V1 criterion not met, n (%)	V2 criterion met, n (%)	V2 criterion not met, n (%)
Detection Test (DET)	97 (95.1)	5 (4.9)	63 (94.0)	4 (6.0)
Identification Test (IDN)	98 (96.1)	4 (3.9)	67 (100.0)	0 (0.0)
One Card Learning Test (OCL)	96 (94.1)	6 (5.9)	59 (88.1)	8 (11.9)
One Back Test (ONB)	101 (99.0)	1 (1.0)	66 (98.5)	1 (1.5)

Note: Data are presented as number (n) and percentage (%) of all participants assessed at the corresponding visit (V1, N=102; V2, N=67). Administrations that yielded a numerical score but failed to meet the relevant integrity

criteria were retained in the primary analyses. Because more than 10% of OCL administrations failed to meet the integrity criterion, all analyses involving OCL and OCL-derived composites were repeated after excluding the failed administrations.

Table S3. Sensitivity analysis of OCL-related outcomes at Visit 2 after exclusion of administrations not meeting the integrity criterion

Analysis	Primary analysis	Sensitivity analysis	Interpretation
OCL analytical sample, n	66	59	Seven participants with a numerical OCL result were additionally excluded on the basis of integrity; one participant had no numerical OCL result
OCL z-score, mean ± SD	0.416 ± 1.217	0.681 ± 0.991	Mean OCL performance was higher after integrity filtering
OCL performance by age group	$t(64) = 2.539$; $p = 0.014$; $d = 0.680$	$t(57) = 2.576$; $p = 0.013$; $d = 0.741$	The significant age-group difference was retained, with a slightly larger effect size
OCL performance by sex	$p = 0.515$	$p = 0.891$	No significant difference in either analysis
OCL performance by baseline complaint count	$p = 0.230$	$p = 0.560$	No significant difference in either analysis
Categorically impaired OCL performance, n/N (%)	7/66 (10.6)	0/59 (0.0)	The primary estimate of categorical OCL impairment should be interpreted cautiously

Note: The sensitivity analysis was performed because eight of OCL administrations failed to meet the predefined CogState integrity criterion. The primary analysis retained all administrations that yielded a numerical score (N=66, missing data n=1), whereas the sensitivity analysis excluded all administrations not meeting the OCL integrity criterion. Cognitive impairment was defined as age-adjusted OCL z-score ≤−1.5. Analyses involving the Learning/Working Memory composite and the global cognitive composites were also repeated after integrity filtering. Their direction and statistical intepretation remained unchanged.

Table S4. Spearman correlations between cognitive performance and selected clinical and demographic variables at Visits 1 and 2

A. Visit 1

Cognitive outcome	N	Age, ρ; *p*	Time since infection, ρ; *p*	Baseline complaint count, ρ; *p*
GMLT z-score	102	0.104; 0.298	−0.079; 0.427	−0.021; 0.837
CPAL z-score	101	**−0.273; 0.006**	0.055; 0.580	−0.110; 0.273
Attention/Psychomotor Function	102	−0.074; 0.457	0.090; 0.369	−0.153; 0.124
Learning/Working Memory	101	0.012; 0.903	0.102; 0.310	−0.076; 0.448
Global Cognitive Index—Standard	101	−0.046; 0.650	0.142; 0.156	−0.155; 0.122
Global Cognitive Index—Exploratory	100	**−0.192; 0.056**	0.083; 0.410	−0.166; 0.098

B. Visit 2

Cognitive outcome	N	Age, ρ; *p*	Time since infection, ρ; *p*	Baseline complaint count, ρ; *p*
GMLT z-score	67	−0.027; 0.825	0.154; 0.213	0.127; 0.306
CPAL z-score	66	**−0.347; 0.004**	−0.087; 0.486	−0.097; 0.440
Attention/Psychomotor Function	67	−0.050; 0.685	−0.039; 0.754	0.070; 0.571
Learning/Working Memory	66	−0.064; 0.612	0.073; 0.562	0.075; 0.548
Global Cognitive Index—Standard	66	−0.054; 0.665	0.008; 0.951	0.114; 0.364
Global Cognitive Index—Exploratory	66	−0.201; 0.106	0.000; 0.999	0.054; 0.665

Note: Presented in the tables are the Spearman's rank coefficients (ρ) with the corresponding two-sided *p*-values. Complaint count was assessed only at baseline; therefore, correlations with Visit 2 cognitive outcomes represent associations with baseline complaint burden rather than concurrently assessed complaints. Statistically significant results are shown in bold.

Table S5A. Multiple linear regression models for cognitive performance at Visits 1 and 2

Cognitive outcome	Visit	N	R^2	Adjusted R^2	F (df)	Model *p*
GMLT z-score	V1	102	0.077	0.039	2.015 (4, 97)	0.098
	V2	67	0.054	−0.007	0.891 (4, 62)	0.475
CPAL z-score	V1	101	0.139	0.103	3.868 (4, 96)	**0.006**
	V2	66	0.124	0.066	2.155 (4, 61)	0.085
Attention/Psychomotor Function	V1	102	0.094	0.057	2.514 (4, 97)	**0.046**
	V2	67	0.045	−0.016	0.735 (4, 62)	0.571
Learning/Working Memory	V1	101	0.090	0.053	2.387 (4, 96)	0.056
	V2	66	0.012	−0.053	0.182 (4, 61)	0.947
Global Cognitive Index—Standard	V1	101	0.136	0.100	3.771 (4, 96)	**0.007**
	V2	66	0.008	−0.057	0.124 (4, 61)	0.973
Global Cognitive Index—Exploratory	V1	100	0.124	0.087	3.350 (4, 95)	**0.013**
	V2	66	0.038	−0.025	0.598 (4, 61)	0.665

Note: Separate multiple linear regression models were fitted at each visit. All models included age, sex, presence of baseline persistent complaints, and time since infection as predictors. R^2 represents the proportion of variance explained by the model, whereas adjusted R^2 accounts for the number of predictors included. Statistically significant overall models are shown in bold.

Table S5B. Predictor coefficients from multiple linear regression models of cognitive performance at Visits 1 and 2

Panel A. Visit 1

Cognitive outcome	Predictor	B	SE	Standardized β	95% CI for B	*p*
**GMLT z-score (N = 102)**	Age	0.016	0.007	0.214	0.001 to 0.030	0.034[a]
	Time since acute infection	−0.015	0.010	−0.145	−0.036 to 0.005	0.141
	Sex	−0.008	0.173	−0.005	−0.353 to 0.336	0.961

Cognitive outcome	Predictor	B	SE	Standardized β	95% CI for B	*p*
	Baseline persistent complaints	−0.250	0.166	−0.150	−0.580 to 0.080	0.136
CPAL z-score (N = 101)	Age	−0.083	0.023	−0.349	−0.129 to −0.037	**<0.001**
	Time since acute infection	0.009	0.033	0.025	−0.057 to 0.075	0.789
	Sex	−0.460	0.552	−0.080	−1.556 to 0.636	0.406
	Baseline persistent complaints	−0.126	0.523	−0.023	−1.164 to 0.912	0.811
Attention/Psychomotor Function (N = 102)	Age	−0.002	0.008	−0.019	−0.017 to 0.014	0.845
	Time since acute infection	0.019	0.011	0.165	−0.003 to 0.042	0.092
	Sex	0.317	0.190	0.164	−0.060 to 0.693	0.098
	Baseline persistent complaints	−0.315	0.182	−0.172	−0.676 to 0.046	0.086
Learning/Working Memory (N = 101)	Age	0.007	0.007	0.092	−0.007 to 0.020	0.356
	Time since acute infection	0.009	0.010	0.084	−0.011 to 0.028	0.388
	Sex	0.317	0.164	0.190	−0.009 to 0.643	0.057
	Baseline persistent complaints	−0.308	0.157	−0.195	−0.621 to 0.004	0.053
Global Cognitive Index—Standard (N = 101)	Age	0.001	0.006	0.021	−0.011 to 0.014	0.829
	Time since acute infection	0.016	0.009	0.170	−0.002 to 0.033	0.078
	Sex	0.323	0.144	0.216	0.038 to 0.608	**0.027**
	Baseline persistent complaints	−0.318	0.138	−0.224	−0.591 to −0.044	**0.023**
Global Cognitive Index—Exploratory (N = 100)	Age	−0.010	0.007	−0.144	−0.023 to 0.003	0.144
	Time since acute infection	0.006	0.009	0.064	−0.013 to 0.025	0.509
	Sex	0.353	0.157	0.219	0.041 to 0.665	0.027
	Baseline persistent complaints	−0.245	0.149	−0.162	−0.540 to 0.051	0.103

Panel B. Visit 2

Cognitive outcome	Predictor	B	SE	Standardized β	95% CI for B	*p*
GMLT z-score (N = 67)	Age	0.002	0.009	0.033	−0.015 to 0.020	0.802

Cognitive outcome	Predictor	B	SE	Standardized β	95% CI for B	*p*
	Time since acute infection	0.020	0.014	0.184	−0.007 to 0.047	0.147
	Sex	0.208	0.221	0.118	−0.235 to 0.651	0.351
	Baseline persistent complaints	0.067	0.211	0.041	−0.354 to 0.488	0.752
**CPAL z-score (N = 66)**	Age	−0.058	0.022	−0.331	−0.102 to −0.014	0.011[a]
	Time since acute infection	−0.005	0.034	−0.016	−0.072 to 0.063	0.894
	Sex	−0.203	0.548	−0.045	−1.298 to 0.892	0.712
	Baseline persistent complaints	−0.271	0.521	−0.065	−1.313 to 0.771	0.605
**Attention/Psychomotor Function (N = 67)**	Age	−0.007	0.010	−0.093	−0.027 to 0.013	0.480
	Time since acute infection	−0.010	0.016	−0.076	−0.041 to 0.022	0.546
	Sex	−0.374	0.255	−0.185	−0.884 to 0.137	0.149
	Baseline persistent complaints	0.165	0.243	0.088	−0.320 to 0.651	0.499
**Learning/Working Memory (N = 66)**	Age	0.00009	0.010	0.001	−0.020 to 0.020	0.993
	Time since acute infection	0.007	0.015	0.063	−0.023 to 0.038	0.629
	Sex	0.156	0.249	0.081	−0.341 to 0.653	0.533
	Baseline persistent complaints	−0.083	0.237	−0.047	−0.557 to 0.390	0.725
**Global Cognitive Index—Standard (N = 66)**	Age	−0.004	0.008	−0.071	−0.020 to 0.011	0.597
	Time since acute infection	−0.001	0.012	−0.009	−0.025 to 0.023	0.944
	Sex	−0.103	0.193	−0.069	−0.490 to 0.283	0.595
	Baseline persistent complaints	0.035	0.184	0.025	−0.333 to 0.403	0.850
**Global Cognitive Index—Exploratory (N = 66)**	Age	−0.012	0.008	−0.195	−0.028 to 0.004	0.144
	Time since acute infection	0.002	0.012	0.020	−0.023 to 0.026	0.874
	Sex	−0.070	0.200	−0.045	−0.469 to 0.330	0.728
	Baseline persistent complaints	−0.009	0.190	−0.006	−0.389 to 0.371	0.962

Note: Separate simultaneous multiple linear regression models were fitted for each cognitive outcome and visit. Age, sex, time since infection, and presence of baseline persistent complaints were entered simultaneously. Complaint status was assessed at baseline (V1) only and was treated as a baseline predictor for Visit 2. B represents the unstandardized regression coefficient. SE is Standard error. Β is the standardized regression

coefficient, CI – the confidence interval. Intercepts are omitted for readability. Variance inflation factors ranged from 1.001 to 1.111.

[a] The age coefficient reached statistical significance in the GMLT model at Visit 1 and the CPAL model at Visit 2. However, the corresponding overall models were not statistically significant – GLMT V1: $F(4,97) = 2.015$, $p = 0.098$; CPAL V2: $F(4,61) = 2.155$, $p = 0.085$. Therefore, these coefficients should be interpreted cautiously.

Table S6. Sensitivity analyses examining the influence of the exact inter-visit (V1 –V2) interval on longitudinal cognitive change

Panel A. Spearman correlations between the exact inter-visit interval and individual cognitive change scores (Δ = V2 − V1)

Cognitive measure	N	Spearman ρ	Unadjusted *p*	BH-FDR q
DET	67	0.254	0.038	0.095
IDN	67	0.132	0.289	0.361
OCL	65	0.160	0.202	0.337
ONB	66	0.135	0.279	0.361
GMLT	67	−0.010	0.939	0.939
CPAL	66	−0.080	0.521	0.579
Attention/Psychomotor Function	67	0.256	0.036	0.095
Learning/Working Memory	65	0.231	0.064	0.128
Standard Global Cognitive Composite	65	0.317	0.010	0.095
Extended Global Cognitive Composite	65	0.274	0.027	0.095

Panel B. Repeated-measures sensitivity analyses including exact inter-visit interval as a covariate

Cognitive measure	N	Time × interval F (df)	Unadjusted *p*	Partial η²	BH-FDR q
DET	67	F(1,65) = 4.008	0.049	0.058	0.128
IDN	67	F(1,65) = 1.478	0.229	0.022	0.327
OCL	65	F(1,63) = 0.356	0.553	0.006	0.691
ONB	66	F(1,64) = 3.877	0.053	0.057	0.128
GMLT	67	F(1,65) = 0.004	0.953	<0.001	0.953
CPAL	66	F(1,64) = 0.180	0.673	0.003	0.748
Attention/Psychomotor Function	67	F(1,65) = 3.628	0.061	0.053	0.128
Learning/Working Memory	65	F(1,63) = 2.807	0.099	0.043	0.165
Standard Global Cognitive Composite	65	F(1,63) = 6.239	0.015	0.090	0.128
Extended Global Cognitive Composite	65	F(1,63) = 3.567	0.064	0.054	0.128

Note: Change scores were calculated as Δ = V2 – V1. Therefore, positive values indicate improvement, as all standardized cognitive scores were directionally harmonized and higher values represent better performance. In **Panel A,** Spearman correlation was used to assess the association between the exact inter-visit interval and individual cognitive change scores. In **Panel B,** repeated-measures general linear models included the exact inter-visit interval as a covariate, and the Time × interval interaction tested whether longitudinal change differed according to follow-up duration. Benjamini–Hochberg false discovery rate (BH-FDR) correction was applied separately to the 10 comparisons in each panel. Several associations and interaction effects showed nominal significance ($p < 0.05$) before correction for multiple comparisons. However, none remained statistically significant after BH-FDR correction (all $q \geq 0.095$ in Panel A and $q \geq 0.128$ in Panel B). Because CPAL and the Extended Global Cognitive Composite were analyzed using non-parametric paired tests in the primary longitudinal analysis, the corresponding repeated-measures models were considered sensitivity analyses rather than primary analyses.

Table S7. Benjamini–Hochberg false discovery rate-adjusted results for the principal multiple-comparison analysis families

Analysis family	Cognitive measure	Unadjusted *p*	BH-FDR q	Significant after FDR
**Longitudinal categorical impairment (McNemar tests; m = 10)**	DET	0.711	1.000	No
	IDN	1.000	1.000	No
	OCL	0.453	1.000	No
	ONB	**0.006**	**0.063**	No
	GMLT	0.500	1.000	No
	CPAL	0.167	0.557	No
	Attention/Psychomotor Function	1.000	1.000	No
	Learning/Working Memory	1.000	1.000	No
	Standard Global Cognitive Composite	0.815	1.000	No
	Extended Global Cognitive Composite	0.057	0.285	No
**Longitudinal continuous-score comparisons (paired tests; m = 10)**	DET	0.564	0.705	No
	IDN	0.869	0.869	No
	OCL	0.284	0.473	No
	ONB	0.082	0.205	No
	GMLT	0.224	0.448	No
	CPAL	**0.031**	**0.201**	No
	Attention/Psychomotor Function	0.782	0.869	No
	Learning/Working Memory	**0.042**	**0.201**	No
	Standard Global Cognitive Composite	0.435	0.621	No
	Extended Global Cognitive Composite	0.060	0.201	No
**Baseline complaint-status comparisons of composite scores at V1 (m = 4)**	Attention/Psychomotor Function	**0.047**	**0.047**	**Yes**
	Learning/Working Memory	**0.041**	**0.047**	**Yes**
	Standard Global Cognitive Composite	**0.013**	**0.047**	**Yes**
	Extended Global Cognitive Composite	**0.027**	**0.047**	**Yes**

Note: Benjamini–Hochberg false discovery rate (BH-FDR) correction was applied separately within each analysis family. Statistical significance after correction was defined as $q < 0.05$. Unadjusted p-values correspond to those reported in the main manuscript. After correction, the longitudinal findings did not remain statistically significant, whereas all four baseline complaint–composite associations at V1 remained significant.
